# Novel polysaccharide extracted from *Sipunculus nudus* inhibits HepG2 tumour growth in vivo by enhancing immune function and inducing tumour cell apoptosis

**DOI:** 10.1111/jcmm.16793

**Published:** 2021-07-24

**Authors:** Jie Su, Dengyuan Liao, Yongchang Su, Shuji Liu, Linlin Jiang, Jingna Wu, Zhiyu Liu, Yuping Wu

**Affiliations:** ^1^ Key Laboratory of Cultivation and High‐value Utilization of Marine Organisms in Fujian Province Fisheries Research Institute of Fujian Xiamen China; ^2^ Guangdong Provincial Key Laboratory of Marine Resources and Coastal Engineering Zhuhai Key Laboratory of Marine Bioresources and Environment School of Marine Sciences Sun Yat‐Sen University Guangzhou China

**Keywords:** apoptosis, cytokines, hepatoma, immune function, polysaccharides, *Sipunculus nudus*, structure

## Abstract

A novel polysaccharide was extracted from *Sipunculus nudus* (SNP). The molecular weight (MW) of SNP was determined to be 9223 Da by high‐performance gel permeation chromatography analyses, and the structure of the SNP repeat units was determined to be →3,4‐β‐D‐GlcpNAC (1→ and →4) ‐α‐D‐Glcp (1→ in the ratio of 15:1; →2) ‐α ‐D‐Galp ‐ (1→ as a side chain; and β‐D‐Galp‐(1→ and α‐ D‐Glcp ‐ (1→ as end groups by GC‐MS analysis and NMR assays. The effect of SNP on hepatoma HepG2‐bearing mice was analysed to verify its potential in the clinical treatment of liver cancer. A total of 90 male athymic nu/nu mice were divided into therapeutic and preventive groups and fed with different amounts of SNP. The antitumour effect of SNP on HepG2‐bearing mice and mechanism of such were studied by analysing the tumour size, spleen index, thymus index, immune factors in the blood, tumour apoptosis factors, etc. The results suggest that SNP not only increased the index of immune organs in the body, but also enhanced the secretion of immune factors, including interleukin‐2, interferon gamma and tumour necrosis factor‐alpha in the serum. SNP induced the apoptosis of tumour cells via the mitochondrial apoptosis pathway, which upregulated caspase‐3, caspase‐8, caspase‐9 and BCL2‐associated X, but downregulated B‐cell lymphoma‐2 and vascular endothelial growth factor protein expression. In conclusion, SNP inhibited tumour growth by enhancing immune function and inducing tumour cell apoptosis in HepG2‐bearing mice. Therefore, SNP may be further investigated as a promising candidate for future antitumour drugs.

## 1 INTRODUCTION

Hepatitis B virus (HBV)–associated hepatoma is a major cause of cancer‐related mortality in Asia.^1^ Currently, the treatment of hepatoma includes surgery and chemotherapy, but both have disadvantages.^2^ Therefore, the identification of non‐invasive treatments that may increase the survival of patients with cancer is of interest in hepatoma therapy. Phytochemicals have been traditionally used as a remedy for cancer treatment in several Asian countries.

Many polysaccharides extracted from natural resources have been proved to have effective antitumour, immunomodulation, antioxidation and anti‐inflammatory activities.^3^ For instance, polysaccharides from Chinese wolfberry pollen, *Tetrastigma hemsleyanum*, and silkworm possess excellent antioxidant and antitumour activities.^3^, ^4^, ^5^ *Sipunculus nudus* is widespread in the subtidal zones and sea beds of temperate or tropical seas. *Sipunculus nudus* has also long been used as traditional Chinese medicine in folk remedies for the treatment of carbuncles, chronic diarrhoea, nocturia, regulating the functions of the stomach and spleen and for restoring health in debilities caused by various pathogens and ageing.^6^ In vivo and in vitro studies showed that SNP administration may enhance immune function.^7^ In our previous study, SNP was found to exert pro‐apoptotic activity and anti‐HBV activity on HepG2.2.15 cells.^8^ However, the molecular weight and structure of SNP was unclear, and whether SNP has an antitumour effect on animal tumour tissue remained unknown. Therefore, the molecular weight and the structure of SNP was analysed by high‐performance gel permeation chromatography (HPGPC), GC‐MS and NMR assay, and the hepatoma HepG2‐bearing mice model was established to investigate the various biological activities of SNP in vivo. The results demonstrated that SNP, a novel polysaccharide extracted from *Sipunculus nudus*, stimulates the immune function and induces cellular apoptosis to mediate its antitumour activity.

## 2 MATERIALS AND METHODS

### 2.1 Isolation and purification of the polysaccharides

To extract polysaccharides from *Sipunculus nudus*, we followed a previously described method.^8^ *Sipunculus nudus* (diameter 6 ± 2 mm, length 10 ± 2 cm) was collected from the Xiamen market, China. Fresh worms were washed, oven‐dried and crushed into powder. The powder was hydrolysed by trypsin at 50℃ for 5 h. The insoluble material was removed by centrifugation. The supernatant liquor was deproteinized four times using the Sevage method. Then, four volumes of cold ethanol were added to precipitate the material after standing at 4℃ overnight. Then, resulting precipitate was centrifuged (3000 × *g*, 10 min). After washing with ethanol three times, the precipitate was freeze‐dried in vacuo and ground into a powder to produce a crude product. The crude product was subjected to a diethylaminoethyl (DEAE)‐Sepharose anion exchange column (3.0 × 40 cm) eluting at 0.5 ml/min successively with 0.0175 M pH 6.7 phosphate‐buffered solution. Each fraction was collected with 2 ml of elute. The main elution fraction containing the carbohydrates was concentrated, dialysed and lyophilized. For polar separation, polysaccharides were distilled with water and purified by a polysaccharide gel purification system (BoRui sugar Biotechnology Co., Ltd.). Combined with refractive index detector (RI‐502, SHODEX), polysaccharides with symmetrical peaks were detected online. The collected liquid was concentrated by freeze evaporation and freeze‐dried (SNP).

### 2.2 Molecular weight determination

The molecular weight and molecular weight distribution and purity of the polysaccharopeptide were analysed by HPGPC. We used 100 mg of polysaccharide, added 50 ml water for dissolution, centrifuged at 5000 rpm, placed 40 ml of the supernatant into a beaker and then added 60 ml ethanol to supernatant, shook well, placed it at 4℃ refrigerator for 1 h, 0.45‐µm membrane filtered, and the precipitate was dried at 80℃ oven for 4 h. An appropriate amount of the dry precipitate was used, and the solution containing about 10 mg per 1 ml was dissolved using the mobile phase. Then, 10 µL was injected into an HPLC to record the chromatogram. We used GPC software to process the data. The samples and the standard (5000, 11,600, 23,800, 48,600, 80,900 148,000, 273,000, 409,800 and 667,800, all from Sigma) were precisely weighed and prepared into a 10 mg/ml solution, centrifuged at 12,000 rpm for 10 min, the supernatant was filtered through a 0.45 µm microporous membrane, and the samples were then transferred to a 1.8‐ml injection flask.

The column set was a Shodex 805–804 gel column (8 × 300 mm). The eluent was 0.02 M NaAc, with a flow rate of 0.5 ml/min, column temperature of 50℃ and injection volume of 10 μl. The detector was a G1362A 1260 RID.

### 2.3 Structural Analysis of Polysaccharide Bond

We took 10 mg of polysaccharide, added 1 ml water for dissolution, added 1 ml of 100 mg/ml carbodiimide and reacted for 2 h. Then, we added 1 ml of 2 M imidazole; divided the sample into two parts; added either 1 ml of 30 mg/ml NaBH_4_ or 1 ml of 30 mg/ml NaBD_4_ to the parts, separately; and reacted for 3 h. We added 100 μl acetic acid to terminate the reaction. After dialysis, the samples were freeze‐dried and methylated. We added 500 μl DMSO to the lyophilized sample for dissolution, and then, 1 mg NaOH was added and incubated for 30 min. We added 50 μl iodomethane solution to react for 1 h. We added 1 ml water and 2 ml dichloromethane, then vortexed, centrifuged at 5000 rpm and discarded the aqueous phase. We repeated the washing three times. The lower dichloromethane phase was absorbed and evaporated to dryness. We added 100 μl 2 M trifluoroacetic acid (TFA) and reacted at 121℃ for 90 min. Solution was steam‐dried at 30℃. We then added 50 μl 2 m ammonia and 50 μl 1 M NaBD4 to solution, mixed and reacted for 2.5 h at room temperature. We added 20 μl acetic acid to terminate the reaction, dried with nitrogen, washed with 250 μl methanol twice and dried with nitrogen again. We added 250 μl acetic anhydride, and the reaction was carried out at 100℃ for 2.5 h. We added 1 ml water and let solution stand for 10 min. We added 500 μl dichloromethane to solution, vortexed, centrifuged at 5000 rpm and discarded the water phase. We repeated washing three times, then took the lower dichloromethane phase and tested it on a GC‐MS system (Agilent Technologies Inc.).

The chromatographic parameters were as follows: an Agilent 7890A (Agilent Technologies) was used as the chromatographic system. According to the properties of the compounds, the injection volume was 1 μl, the split ratio was 10:1, and the carrier gas was high‐purity helium. The initial temperature of the column incubator was 140℃ for 2.0 min, and the temperature was raised to 230℃ at a rate of 3℃/min for 3 min.

The mass spectrum parameters were as follows: a quadrupole mass spectrometry system (Agilent 5977B; Agilent Technologies) was used in the mass spectrometry system, which was equipped with an electron bombardment ion source (EI) and Mass Hunter workstation. The analytes were detected by electron bombardment ion source (EI) in scan mode with a mass scanning range (m/z) of 30–600.

### 2.4 NMR Analysis

The method of NMR analysis in Shi et al. was followed.^9^ Briefly, SNP (30 mg) dissolved in D_2_O (99.8%) was lyophilized to exchange hydrogen with deuterium three times. The samples were finally dissolved in 0.5 ml D_2_O (99.9%) and transferred into a 5‐mm nuclear tube. The ^1^H, ^13^C and distortionless enhancement by polarization transfer (DEPT‐135) NMR spectra were recorded at 400.13 and 100.61 MHz on a Bruker Avance 400 MHz NMR spectrometer (Bruker) at around 295 K. Two‐dimensional experiments, including the homonuclear 1H/1H correlation spectroscopy (COSY), heteronuclear single‐quantum coherence (HSQC), heteronuclear multiple‐bond correlation (HMBC) and nuclear Overhauser effect spectroscopy (NOESY), using the standard Bruker pulse sequence were also conducted for further analyses. The 1D NMR spectra were recorded by suppressing the ^1^H and ^13^C signals at 2.15 and 30.2 ppm of acetone, respectively.

### 2.5 Cytotoxicity evaluation

Cell counting kit (CCK)‐8 was used for cytotoxicity evaluation of SNP. Human hepatocyte cells (LO**_2_**) were first counted, and approximately 10^4^ cells per well were seeded in a 96‐well cell culture plate (Corning Inc.). Then, after incubation at 37℃ in a humidified atmosphere with 5% CO_2_ for 12 h, the culture medium was replaced by a series of concentrations of SNP (0.0, 0.25, 0.5, 1.0, 2 mg/ml) diluted with the corresponding culture fluid. The culture medium (containing SNP of different concentrations) was replaced every 2 days. After 7 days of culture, 10 μl of the CCK‐8 reagent (Beyotime C0037) was added into each well, and OD at 450 nm was measured using a multifunction microplate reader (PerkinElmer VICTOR Nivo) after incubation for 2 h at 37℃. Five replicates were made for each measurement.

### 2.6 Tumour xenotransplantation

Male athymic nu/nu mice (age, 6–8 weeks; weight, 18–22 g) were purchased from Shanghai SLAC Laboratory Animal Co., Ltd. Human HepG2 cells were obtained from Shanghai MeiXuan Biological Technology Co., Ltd. The short tandem repeat (STR) profile analysis indicated that the HepG2 cells were hepatoblastoma cells.

HepG2 cells supplemented with 100 μl Dulbecco's modified Eagle medium (DMEM, Thermo Fisher Scientific, Inc.) were injected into the right upper flank region of the mice (2 × 10^6^ cells per mouse). The mice were screened for experiments when the size of the tumour was measurable. All animals were housed with free access to food and water in plastic cages at 21 ± 2˚C with 30–70% relative humidity and maintained on a 12‐h light/dark cycle. All procedures followed the guidelines for humane treatment of animals set by the Association of Laboratory Animal Sciences and the Center for Laboratory Animal Sciences at Xiamen University. This study was performed from May 2017 to May 2018 and was approved by the Animal Ethics Committee of Xiamen University (approval date 13 March 2017).

### 2.7 Experimental design

A total of 90 athymic nu/nu mice were used in this study, which were divided into three groups: treatment, prevention and positive groups. For the treatment experiment (marked as a), 40 mice were inoculated with tumour cells. When the tumours reached millet size, mice were screened as treatment animal models (marked as a). These animals were randomly divided into four groups (*n* = 6 mice/group): vehicle control group (saline, 0.2 ml/mouse, referred to as ^a^Ctrl) and three treatment groups at various dosages (SNP at 50, 100 and 200 mg/kg, referred to as ^a^SNP‐50, ^a^SNP‐100 and ^a^SNP‐200, respectively). For the preventive experiment (marked as b), another 40 mice were randomly divided into four groups: a vehicle control group (saline, 0.2 ml/mouse, referred to as ^b^Ctrl) and three precondition groups at various dosages (SNP at 50, 100 and 200 mg/kg, referred to as ^b^SNP‐50, ^b^SNP‐100 and ^b^SNP‐200, respectively). After preconditioning for 1 month, these animals also received tumour xenotransplantation. When the tumours reached millet size, mice were screened as preventive animal models (*n* = 6/group). After the two animal models were established, the drugs were administered intragastrically once daily for 16 consecutive days and samples were collected. Ten mice inoculated with tumour cells were fed with APS after tumour reach millet size as positive control group (Astragalus polysaccharides (APS), 100 mg/kg, referred to as APS). The experiment was carried out in batches.

### 2.8 Sample collection

At the end of the experimentation, the mice were euthanized by the inhalation of CO_2_ at a rate of 10–30% of the volume of the euthanasia chamber per min. The blood samples were collected from orbit for the determination of cytokines. The tumours, thymuses and spleens of all animals were removed and weighed. The tumour volumes were measured and calculated using the following equation: volume = w × ow^2^ / 2, where w is the maximal width (mm) and ow is the maximal orthogonal width (mm). The tumour inhibition rate and organ index were calculated using the following equations: tumour inhibition rate (by weight) = (mean tumour weight of control group – mean tumour weight of treatment group)/mean tumour weight of control group × 100%; tumour inhibition rate (by volume) = (mean tumour volume of control group – mean tumour volume of treatment group) / mean volume of control group × 100%; organ index = organ weight (mg) / body weight (g) × 100%.

### 2.9 Histopathological analysis

Tumour tissues of all mice were fixed in 4% paraformaldehyde solution for 24 h and then embedded into paraffin blocks. Contiguous sections were collected for haematoxylin and eosin (H&E) staining preparation. H&E staining was performed to observe the pathological changes and was performed following standard procedures. Paraffin sections were incubated with xylol twice for 15 min for deparaffination, continuously washed in graded alcohol (70%, 80% and 90%) for 3 min for rehydration and then rinsed with running water for 1 min. The sections were then stained with haematoxylin for 5 min, rinsed with running water for 1 min, rinsed with 0.1% hydrochloric acid for 15 s and then rinsed again with running water for 20 min. Subsequently, the sections were stained with eosin for 5 min and rinsed with running water for 10 min. The sections were then immersed in 70% (45 s), 75% (45 s), 85% (45 s), 90% (45 s) and 100% ethanol (twice for 2 min) for dehydration and then immersed in xylene twice for 2 min to make the section transparent. Then, the slides were mounted on cover glass using cedarwood oil and the samples were imaged under a light microscope for morphological observation.

### 2.10 Immunofluorescence staining

The tissue sections were hydrated, heated in a microwave for 10–15 min with 0.01 mol/l pH 6.0 citrate buffer, cooled for 20–30 min, and then washed three times with phosphate‐buffered saline (PBS). The corresponding antibodies (Cas3, 9664, Cell Signaling Technology (Cst); Cas8, 9496, Cst; Cas9, 20750, Cst; VEGF, Ab1316, Abcam; Bax, Ab53154, Abcam; Bcl‐2, Ab692, Abcam) dilution solution was added and incubated overnight at 4˚C. The dilution ratio of primary antibodies was 1:5000. Sections were washed five times with PBS and incubated with fluorescent‐labelled second antibody (D110061; Sangon Biotech (Shanghai) Co., Ltd.) for 30 min. The dilution ratio of second antibody was 1:1000. Then, sections were washed five times with PBS, incubated with 4’,6‐diamidino‐2‐phenylindole (DAPI) solution for 2 min and washed three more times with PBS. Filter paper was then used to remove residual PBS on the outside the specimens, and sections were sealed with glycerol for fluorescence microscope observation.

### 2.11 ELISA

The blood samples of mice in each group were centrifuged at 1000 ×*g* for 10 min, and the serum was collected. The cytokines, including IL‐2, IFN‐γ and TNF‐α, were detected using the relevant commercial ELISA kits (MEXN‐M0040, MEXN‐M0046 and MEXN‐M0047, respectively, Shanghai Meixuan Biological Science and Technology Ltd.) according to the manufacturer's instructions (Cusabio Technology LLC). The optical density (OD) was measured at 450 nm using an MK3 microplate reader (Thermo Fisher Scientific, Inc.).

### 2.12 Reverse transcription‐quantitative PCR (RT‐qPCR)

Total RNA from tumour samples was extracted using TRIzol^®^ reagent (Invitrogen; Thermo Fisher Scientific, Inc.), and gene expression was analysed via RT‐PCR. The primers were synthesized by Shanghai Shenggong Bioengineering Co., Ltd. as shown in Table S1. cDNA was synthesized using a ReverTra Ace^®^ qPCR RT kit (Toyobo Life Science), followed by qPCR using SYBR Green Real‐Time PCR Master Mix‐Plus (Toyobo Life Science). The expression of β‐actin was used as the internal control.

### 2.13 Western blotting

Tumour tissue (≥50 mg) in each group was harvested, minced and lysed with radioimmunoprecipitation assay (RIPA) lysis buffer (Beijing Solarbio Science & Technology Co., Ltd.). The extracted proteins were quantified using a bicinchoninic acid (BCA) protein assay kit (Beijing Solarbio Science & Technology Co., Ltd.). An equal amount of sample (20 μg protein/lane) was separated via SDS‐PAGE and then transferred onto polyvinylidene fluoride membranes. The membranes were blocked with 5% skim milk for 1 h at 20–25 ˚C and then immunoblotted with indicated antibodies at 4 ˚C overnight. The primary antibodies used were as follows: activating transcription factor 4 (ATF4, 11815s, Cst), DNA damage‐inducible transcript 3 (DDIT3, 5554s, Cst), IκBα, cellular communication network factor 1 (CYR61, 14479s, Cst), heat‐shock protein 90 (HSP90, Ab13495, Abcam), vascular endothelial growth factor (VEGF, 2445s, Cst) and β‐actin (ab6276, Abcam). The dilution ratio of primary antibodies was 1:5000. Subsequently, the membranes were washed three times with Tris‐buffered saline (TBS)‐Tween (TBST) for 5 min each time. Then, membranes were incubated with horseradish peroxidase–conjugated goat anti‐rabbit and mouse polyclonal secondary antibodies (ab7090, ab97040, Abcam) at room temperature. The dilution ratio of secondary antibodies was 1:3000. After 1 h, the membranes were washed three times in TBST for 10 min each time, and then, the protein signals were visualized using a hypersensitive enhanced chemiluminescent kit (Beijing Solarbio Science & Technology Co., Ltd.). The bands were semiquantified using Image‐Pro Plus 6.0 software Media Cybernetics, Inc.), which was used to analyse the protein levels.

### 2.14 Statistical analysis

Data from ≥3 independent experiments are presented as the mean ± standard deviation (SD). Statistical significance of difference was performed with Student's t test and two‐factor without replication (analysis of variance: ANOVA). The data were considered to be significant at *p* < 0.05.

## 3 RESULTS

### 3.1 Molecular weight determination

**FIGURE 1:**
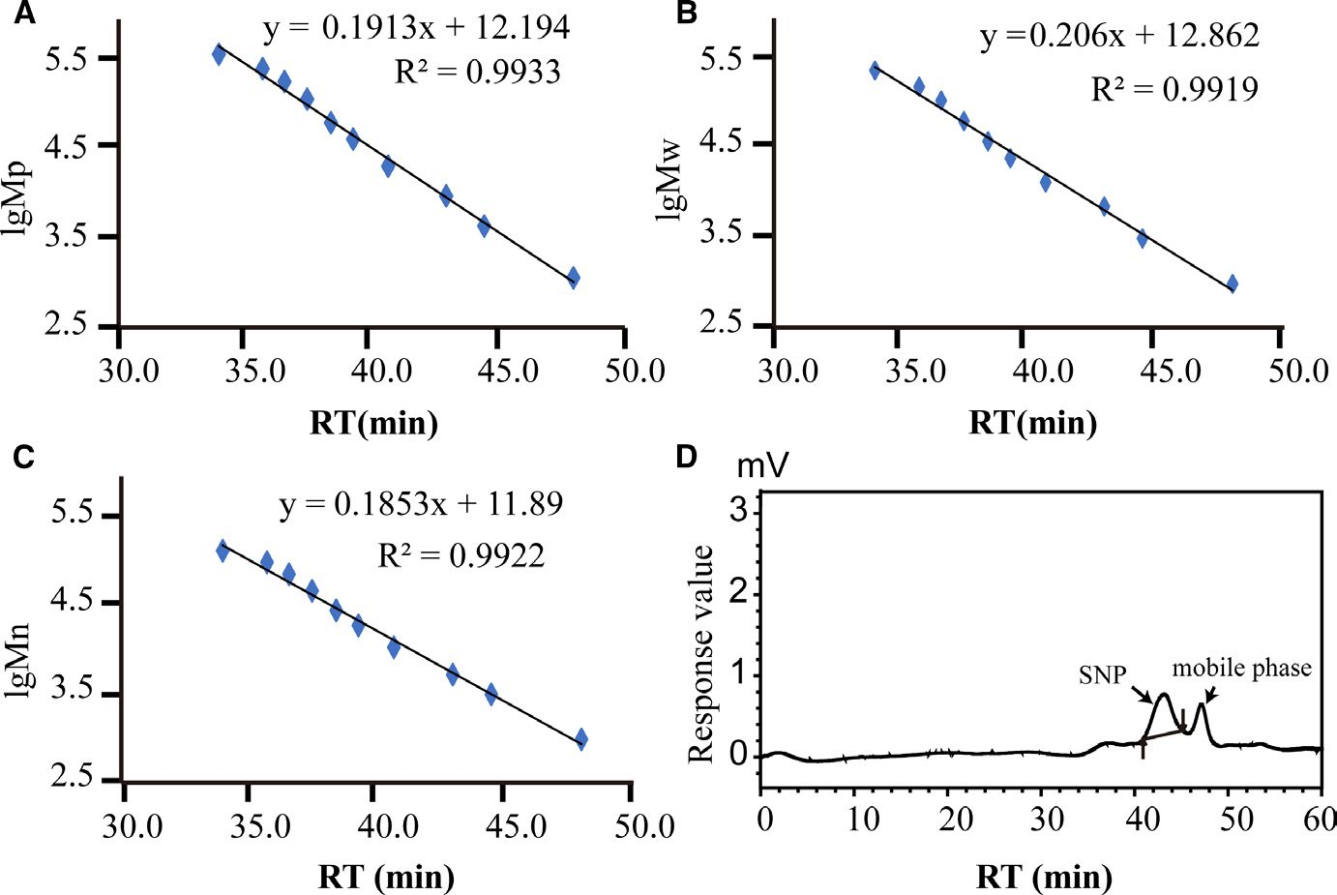
Sipunculus nudus (SNP) molecular weight determination by high‐performance gel permeation chromatography (HPGPC). (A–C) Standard curve adopting the logarithm of polysaccharide standards and retention time; (D) high‐performance gel permeation chromatography of SNP. MP, peak molecular weight; MW, average molecular weight; Mn, number average molecular weight

The molecular weight of SNP was determined by HPGPC. The logarithm of polysaccharide standards and retention time (RT) are plotted in Figure 1A–C. The molecular weight (MW) of SNP is 922 3D (Table 1) according to the RT of SNP (Figure 1D) and standard curve calculating.

**TABLE 1:** SNP molecular weight determination by HPGPC

Sample	RT (min)	lgMp	lgMw	lgMn	Mp	Mw	Mn
SNP	43.190	3.9	4.0	3.9	8546	9223	7707

RT, retention time. The mass average molar mass (Mw), number average molar mass (Mn), molar mass at the peak of the chromatogram (Mp).

### 3.2 Structural analysis of polysaccharide bond

The results of GC‐MS analysis after SNP methylation are shown in Figure S1, and the statistics of quantitative analysis are shown in Table S2. The results showed that there were 16 different molecular bonds, but the main structure was composed of galactose and glucose.

### 3.3 NMR analysis

To obtain the structural information of SNP, one‐dimensional NMR (^1^H‐NMR, ^13^C‐NMR and distortionless enhancement by polarization transfer (DEPT)‐135)) and two‐dimensional NMR (correlation spectroscopy (COSY), heteronuclear single‐quantum coherence (HSQC), heteronuclear multiple‐bond correlation (HMBC) and total correlation spectroscopy (TOCSY)) were used to determine the chemical shifts of the H and C of the main sugar residues and to infer the connection sequence of each sugar residue.

Most of the hydrogen spectra signals of SNP were in the range of δ 3.0–5.5 ppm, and δ 4.5–5.5 ppm is usually the heterohead proton (H‐1) resonance region.^10^ The 1H‐NMR spectrum of SNP is shown in Figure S2. Many proton resonance signals were concentrated in the region of δ 3.0–5.5 ppm. According to the HSQC spectrum (Figure S5) and 1C‐NMR spectrum (Figure S3) of SNP, five main heterohead proton coupling signals were found in the heterohead region. The heterohead proton signals were δ 5.33, δ 5.23, δ 4.87, δ 4.55 and δ 4.34 ppm, indicating that there might be five monosaccharide residues. According to their chemical shift signal intensity, the corresponding five sugar residues were labelled as A, B, C, D and E, as shown in Figure S2.

For sugar residue A, heterocephalic signals δ 4.34 ppm (H‐1) and δ 105.86 ppm (C‐1) indicated that residue A was in β configuration. According to the chemical shift δ 4.34 ppm of H‐1 determined by 1H NMR, the signals of H‐2, H‐3, H‐4 and H‐5 were identified by the COSY spectrum (Figure S4). The chemical shifts of H‐2, H‐3, H‐4 and H‐5 of sugar residue A were assigned to δ 3.56, δ 3.68, δ 3.96 and δ 3.66 ppm, respectively, and one of the chemical shifts of H on position 6 was assigned to δ 3.77 ppm. After the assignment of the chemical shifts of hydrogen on the sugar ring, the chemical shifts of each carbon on the sugar ring were assigned by HSQC correlation spectrum (Figure S5), which were δ 105.86, δ 75.40, δ 75.50, δ 72.19, δ 74.24 and δ 63.43 ppm (Table S3). Combined with the methylation results and literature reports,^9^, ^11^, ^12^ we inferred that sugar residue A was β‐ D‐Galp ‐ (1→).

For sugar residue B, heterocephalic signals δ 5.23 ppm (H‐1) and δ 98.81 ppm (C‐1) indicated that residue B was in α configuration. According to the H‐1 chemical shift δ 5.27 ppm of sugar residue B determined by 1H NMR, the H‐2, H‐3, H‐4 and H‐5 signals were identified by the COSY spectrum (Figure S4). The chemical shifts of H‐2, H‐3, H‐4 and H‐5 of sugar residue B were assigned to δ 3.91, δ 4.09, δ 3.93 and δ 3.51 ppm, respectively. The H‐6a signal was assigned to δ 3.68 ppm by the HSQC (Figures S5) correlation spectrum. After the chemical shifts of hydrogen on the sugar ring were assigned, the chemical shifts of each carbon on the sugar ring were assigned by the HSQC correlation spectrum (Figure S5): δ 98.81, δ 75.88, δ 71.11, δ 71.89, δ 71.60 and δ 63.40 ppm (Table S3). The chemical shifts of C‐1 and C‐2 shifted to the low field, indicating that the residues were substituted at C‐1 and C‐2 of the sugar ring. Combined with the methylation results and reports,^13^ we inferred that residue B was →2) ‐ α ‐ D‐Galp ‐ (1→).

For sugar residue C, heterocephalic signals δ 4.87 ppm (H‐1) and δ 100.95 ppm (C‐1) indicated that the residue C was in α configuration. According to the H‐1 chemical shift δ 4.87 ppm of sugar residue C determined by 1H NMR, the H‐2, H‐3, H‐4 and H‐5 signals were identified by the COSY spectrum (Figure. S4). The chemical shifts of H‐2, H‐3, H‐4 and H‐5 of sugar residue C were assigned to δ 3.47, δ 3.66, δ 3.32 and δ 3.88 ppm, respectively. The H‐6a signal was attributed as inferred from HSQC (Figure. S5): δ 3.75 ppm. After the assignment of the chemical shifts of hydrogen on the sugar ring, the chemical shifts of each carbon on the sugar ring were assigned using the HSQC correlation spectrum (Figure S5), which were δ 100.95, δ 71.60, δ 72.19, δ 72.27, δ 69.17 and δ 63.30 ppm (Table S3). The chemical shift of C‐1 shifted to low field, indicating that the residue was substituted at the C‐1 position of the sugar ring. Combined with the methylation results and prior reports,^14^, ^15^ we inferred that the residue C was α‐D‐Glcp ‐ (1→).

For sugar residue D, heterocephalic signals δ 5.33 ppm (H‐1) and δ 100.33 ppm (C‐1) indicated that residue D was in α configuration. According to the H‐1 chemical shift δ 5.33 ppm of sugar residue C determined by 1H NMR, the H‐2, H‐3, H‐4 and H‐5 signals were identified by the cross peak of COSY (Figure S4). The chemical shifts of H‐2, H‐3, H‐4 and H‐5 of sugar residue C were assigned to δ 3.44, δ 3.97, δ 3.65 and δ 3.34 ppm, respectively. The assignment of H‐6a signal was inferred from HSQC (Figure S5), which was δ 3.69 ppm. After the assignment of the chemical shifts of hydrogen on the sugar ring, the chemical shifts of each carbon on the sugar ring were assigned using the HSQC correlation spectrum (Figure S5), which were δ 100.33, δ 69.55, δ 71.60, δ 76.11, δ 71.81 and δ 63.45 ppm (Table S3). The chemical shift of C‐4 shifted to the low field, which indicated that the residue was substituted at the C‐4 position of the sugar ring. Combined with the methylation results and reports in the literature,^9^, ^11^, ^12^, ^13^, ^14^, ^15^ we inferred that the residue D was →4) ‐α‐ D‐Glcp ‐ (1→).

For sugar residue E, heterocephalic signals δ 4.55 ppm (H‐1) and δ 104.18 ppm (C‐1) indicated that the residue E was in β configuration. According to the H‐1 chemical shift δ 4.55 ppm of sugar residue C determined by 1H NMR, the H‐2, H‐3, H‐4 and H‐5 signals were identified by COSY (Figure S4) and the literature.^9^, ^11^, ^12^, ^13^, ^14^, ^15^ The H‐2, H‐3, H‐4 and H‐5 chemical shifts of sugar residue C were assigned to δ 3.74, δ 3.55, δ 3.44 and δ 3.44 ppm, respectively. After the chemical shifts of hydrogen on the sugar ring were assigned, the chemical shifts of each carbon on the sugar ring were assigned using the HSQC correlation spectrum (Figure S5), which were δ 104.18, δ 57.40, δ 80.31, δ 83.33, δ 73.36 and δ 63.42 ppm (Table S3). The chemical shifts of C‐4 and C‐3 shifted to the low field, indicating that the residues were substituted at the C‐4 and C‐3 positions of the sugar ring. Combined with findings of previous reports,^16^, ^17^ we inferred that the residue E was →3,4) ‐ β ‐ D‐Glcpnac (1→).

Based on the coupling signals of heterocephalic hydrogen with carbon or heterocephalic carbon with hydrogen in the HMBC remote correlation spectrum, we further inferred the interconnection sequence of each sugar residue. The HMBC correlation spectrum of SNP is shown in Figure S6, from which the following can be found: C‐1 (δ 105.86 ppm) of residue A and H‐4 (δ 3.44 ppm) of residue E have a coupling signal (A C‐1/E H‐4). The NOESY spectrum of the SNP sample is shown in Figure S7, from which the following can be found: residue A H‐2 (δ 3.56 ppm) and residue D H‐1 (δ 5.33 ppm) have coupling signals (D H‐1/A H‐2). There is a coupling signal (B H‐1/ C H‐5) between residue B H‐1 (δ 5.23 ppm) and residue C H‐5 (δ 3.88 ppm). There is a coupling signal (E H‐1/B H‐2) between residue E H‐1 (δ 4.55 ppm) and residue B H‐2 (δ 3.56 ppm). In the H spectrum of SNP, the signal peaks of E and A were integrated, and the area ratio was about 3:2. The results of demethylation analysis showed that the molar ratio of residues E, A, B, C and D was about 15:10:5:5:1.

**FIGURE 2:**
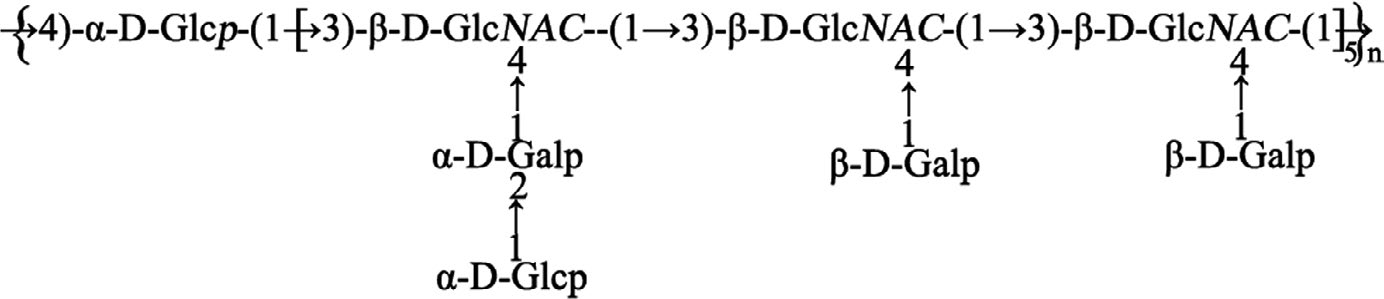
The repeating units of SNP

Based on the analysis of the methylation results (Table S3) and the one‐ and two‐dimensional NMR information, the preliminary structure of the SNP was deduced to be a type of polysaccharide with the main chain composed of →3,4)‐β‐D‐GlcpNAC (1→ and →4) ‐α‐D‐Glcp (1→ in the ratio of 15:1; →2) ‐α ‐D‐Galp ‐ (1→ as a side chain, β‐D‐Galp‐(1→; and α‐ D‐Glcp ‐ (1→ as the end group. The possible structural repeating units are shown in Figure 2.

### 3.4 SNP suppresses tumour growth in HepG2 tumour‐bearing mice

**FIGURE 3:**
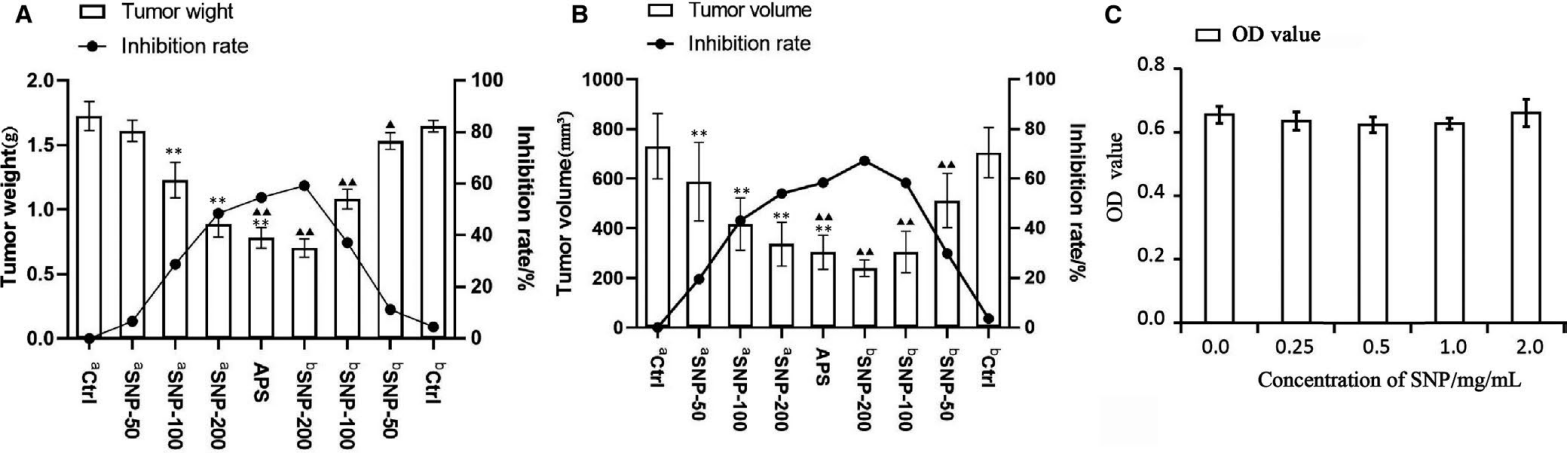
Antitumour activity of SNP in HepG2 tumour‐bearing mice. A, Effect of SNP on tumour weight and inhibition rate (determined using weight). B, Effect of SNP on tumour volume and inhibition rate (determined using volume). C, Cytotoxicity evaluation of SNP on hepatocyte LO2 cells after 7 days culture. a, the therapeutic experiment; b, the preventive experiment. Data are presented as the mean ± SD (*n* = 6). **p < 0.01 vs. aCtrl group; ^▲▲^ *p* < 0.01 vs. bCtrl group. Ctrl, control; SNP, Sipunculus nudus polysaccharide; APS, astragalus polysaccharides

The tumour weight and volume were measured, and the corresponding inhibition rates were calculated simultaneously. In the therapeutic experiment, the tumour weights of the ^a^SNP‐100 and ^a^SNP‐200 groups were significantly lower compared with those of the ^a^Ctrl group (both *p* < 0.01; Figure 3A, left). The tumour volumes of all SNP‐treated groups were significantly smaller compared with those of the ^a^Ctrl group (all *p* < 0.01; Figure 3B, left). These results suggested that SNP has a certain therapeutic effect on the tumours of HepG2 tumour‐bearing mice. In the preventive experiment, the tumour weights of all SNP‐related groups were significantly lower compared with the ^b^Ctrl group (all *p* < 0.01; Figure 3A, right). The tumour volumes of the ^b^SNP‐100 and ^b^SNP‐200 groups, but not the ^b^SNP‐50 group, were significantly smaller compared with the ^b^Ctrl group (both *p* < 0.01; Figure 3B, right). These results demonstrated that SNP may have a prophylactic effect on the tumours of HepG2 tumour‐bearing mice.

The tumour inhibition rates (based on weight) that were closest to those of the *Astragalus* polysaccharide (APS)–positive group (54.67%) were those of the ^a^SNP‐200 (48.52%) and ^b^SNP‐200 groups (59.30%) (Figure 3A). The tumour inhibition rates (based on volume) that were similar with the APS‐positive group (58.43%) were those of the ^a^SNP‐200 (53.99%) and ^b^SNP‐100 (58.32%) group, whereas these were significantly decreased in the ^b^SNP‐200 group (67.21%; *p* < 0.01) (Figure 3B). These results indicated that SNP at a high concentration could effectively suppress tumour growth to the same extent or more than the APS‐positive control. Cytotoxicity evaluation of SNP on hepatocyte LO_2_ cells after 7 days culture with different concentration of SNP (0.0, 0.25, 0.5, 1.0 and 2.0 mg/ml) by cell counting kit (CCK)‐8 assay. The results showed that SNP (0–2.0 mg/ml) had no toxic effect on hepatocyte LO_2_ cells (Figure 3C).

### 3.5 SNP changes the pathological morphology of tumour tissue

**FIGURE 4:**
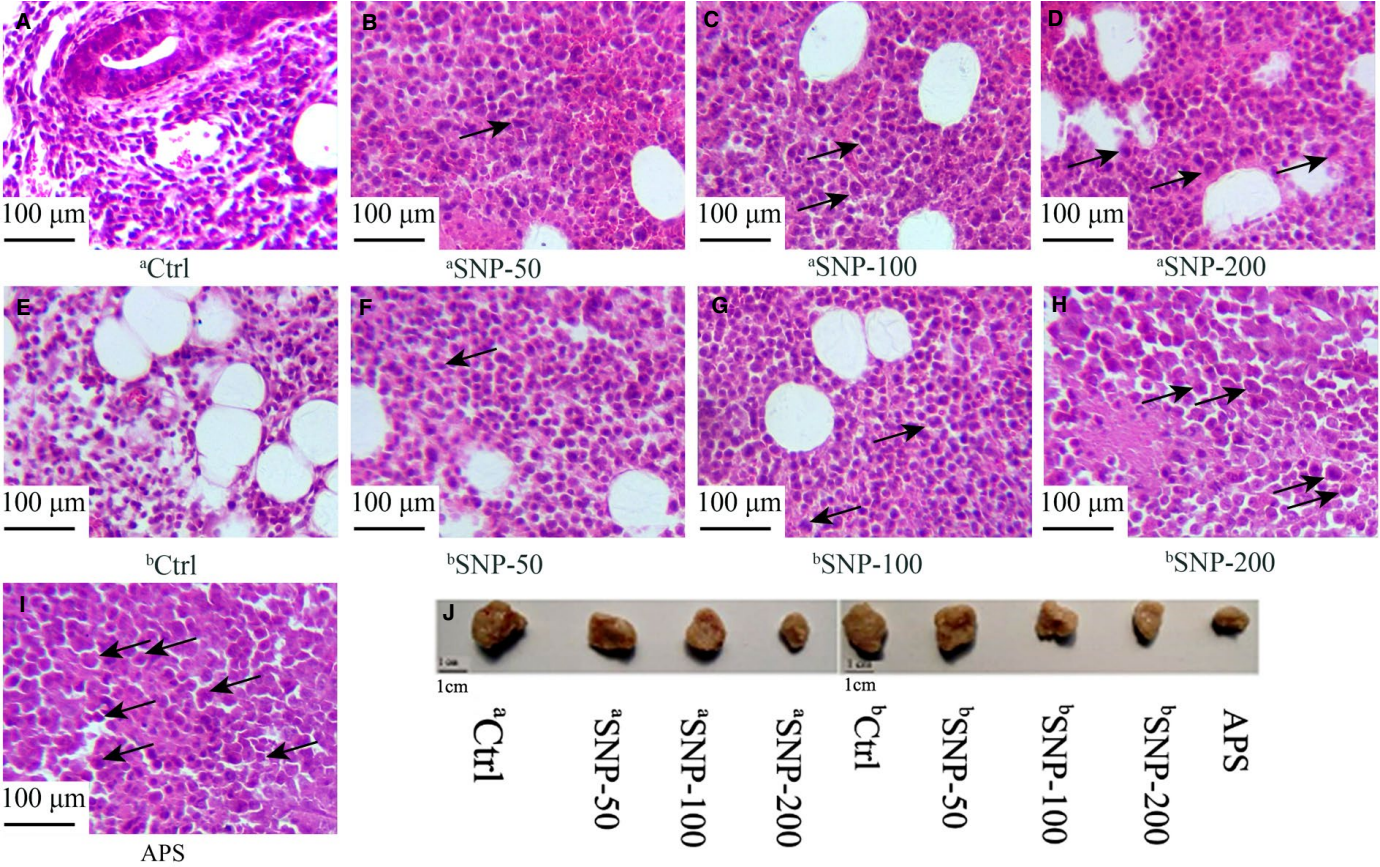
Morphological observation of tumour cells from HepG2 tumour‐bearing mice using haematoxylin and eosin (H&E) staining. A, aCtrl group, vehicle control in the therapeutic experiment. (B) aSNP‐50, (C) aSNP‐100 and (D) aSNP‐200 groups (representing SNP treatments at concentrations of 50, 100 and 200 mg/kg, respectively) in the therapeutic experiment. E, bCtrl group, vehicle control in the preventive experiment. (F) bSNP‐50, (G) bSNP‐100 and (H) bSNP‐200 groups (representing SNP preconditioning at concentrations of 50, 100 and 200 mg/kg) in the preventive experiment. (I) APS group, used as a positive control. J, Images of representative tumours. Ctrl, control; SNP, Sipunculus nudus polysaccharide

Histological analysis of the tumour samples was conducted to evaluate the antitumour efficacy of SNP on the tumours of HepG2 tumour‐bearing mice. Images of representative tumours (Figure 4J) demonstrated that the maximum tumour size observed in this study was 936 mm^3^ in the ^a^Ctrl group. After haematoxylin and eosin (H&E) staining, the tumour cells of the ^a^Ctrl and ^b^Ctrl groups were diffusely distributed and arranged densely with different sizes (Figure 4A,E). However, the tumour cells notably exhibited various histological features in all SNP‐related groups (Figure 4B–D, F–H), such as loose arrangement, nuclear shrinkage, large areas swollen and necrosis, which were the same as those observed in the APS‐positive control group (Figure 4I). There were visible deformed tumour cells in all SNP‐related groups (indicated by the black arrows), suggesting that SNP enhanced the tumour cell apoptosis.

### 3.6 SNP increases the indices of the immune organs (thymus and spleen)

**FIGURE 5:**
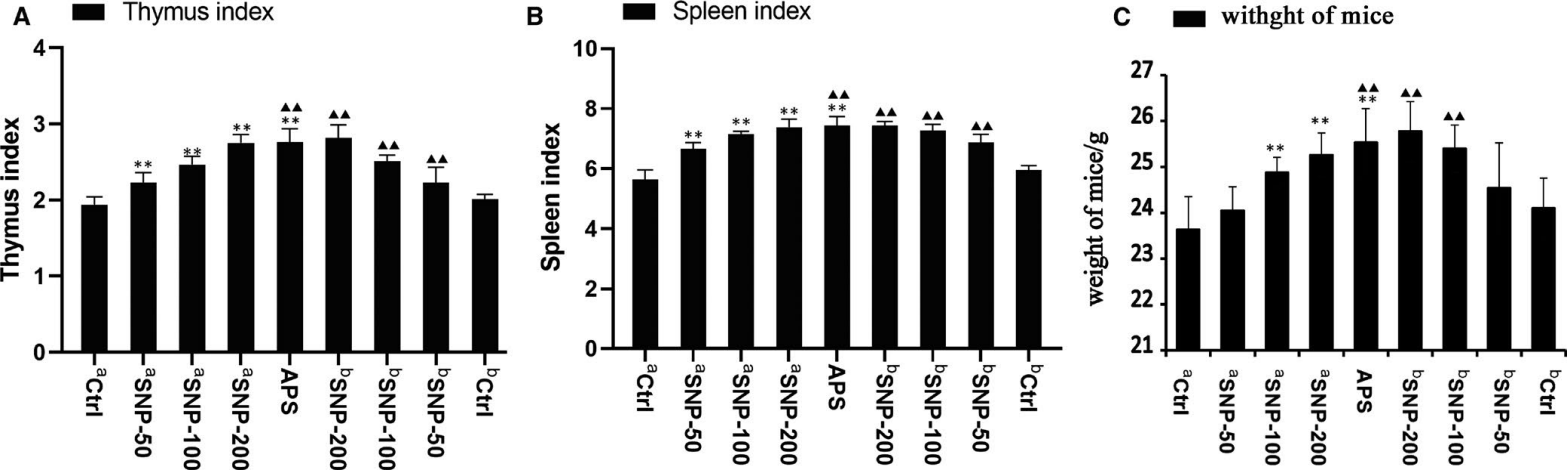
Effects of SNP on the thymus and spleen indices of HepG2 tumour‐bearing mice. (A) Thymus indices and (B) spleen indices were calculated as the ratio of the spleen or thymus weight (mg) to the body weight (g) of each mouse. (C) The weight of mice. a, therapeutic experiment; b, preventive experiment. Data are presented as the mean ± SD (*n* = 6). **p < 0.01 vs. aCtrl group; ▲▲*p* < 0.01 vs. bCtrl group. Ctrl, control; SNP, Sipunculus nudus polysaccharide

The thymus and spleen indices are often used to reflect the immunological function of the organism. Therefore, we evaluated the thymus and spleen indices in the HepG2 tumour‐bearing mice after SNP. The spleen and thymus indices were elevated in all SNP‐related groups (*p* < 0.01; Figure 5A,B). The weight of mice in SNP groups and APS‐positive group increased significantly (Figure 5C). These results indicated that SNP exerted a promoting effect on immunological function. The thymus and spleen indices of the ^a^SNP‐200 and ^b^SNP‐200 groups (high SNP concentration groups) were the same as the APS‐positive group (Figure 5), suggesting that SNP suppressed tumour growth by enhancing the immune function of the mice.

### 3.7 SNP enhances the secretion of cytokines

The antitumour function of SNP may be associated with the immunological reaction. Cytokines are a distinct category of small proteins that are important in immune response and cell signalling. To determine whether SNP influenced the secretion of cytokines, the serum levels of interleukin (IL)‐2, interferon (IFN)‐γ and tumour necrosis factor (TNF)‐α were measured using ELISA.

**FIGURE 6:**
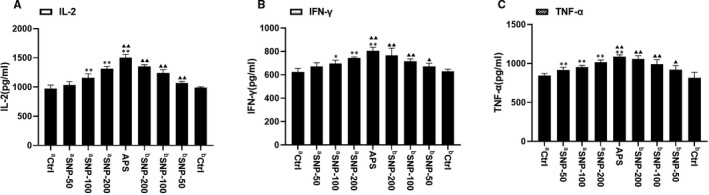
Effect of the SNP on the serum levels of cytokines in HepG2 tumour‐bearing mice as determined using ELISA: (A) interleukin (IL)‐2, (B) interferon (IFN)‐γ and (C) tumour necrosis factor (TNF)‐α levels. a, therapeutic experiment; b, preventive experiment. Data are presented as the mean ± SD (*n* = 6). *p < 0.05, **p < 0.01 vs. aCtrl group; ▲▲*p* < 0.05, ▲▲*p* < 0.01 vs. bCtrl group. Ctrl, control; SNP, Sipunculus nudus polysaccharide

In the therapeutic experiment, the IL‐2 and IFN‐γ levels in the ^a^SNP‐100 and ^a^SNP‐200 groups were significantly upregulated compared with those of the ^a^Ctrl group (both *p* < 0.01), but this was not found in the ^a^SNP‐50 group (Figure 6A,B, left). However, the production of TNF‐α in all SNP‐related groups was upregulated compared with the ^a^Ctrl group (all *p* < 0.01; Figure 4C, left). In the preventive experiment, the secretions of IL‐2, IFN‐γ and TNF‐α in all SNP‐related groups were higher compared with those of the ^b^Ctrl group (*p* < 0.01 and *p* < 0.05 for IFN‐γ and TNF‐α levels of ^b^SNP‐50 group, respectively; Figure 6A–C, right). The levels of IL‐2, IFN‐γ and TNF‐α in the ^a^SNP‐200 and ^b^SNP‐200 groups were similar to those of the APS‐positive group (Figure 6). These data indicated that SNP exerted an antitumour function by upregulating the secretion of cytokines IL‐2, IFN‐γ and TNF‐α.

### 3.8 SNP regulates the expression levels of signalling proteins (ATF4, DDIT3, IκBα, CYR61, HSP90 and VEGF)

**FIGURE 7:**
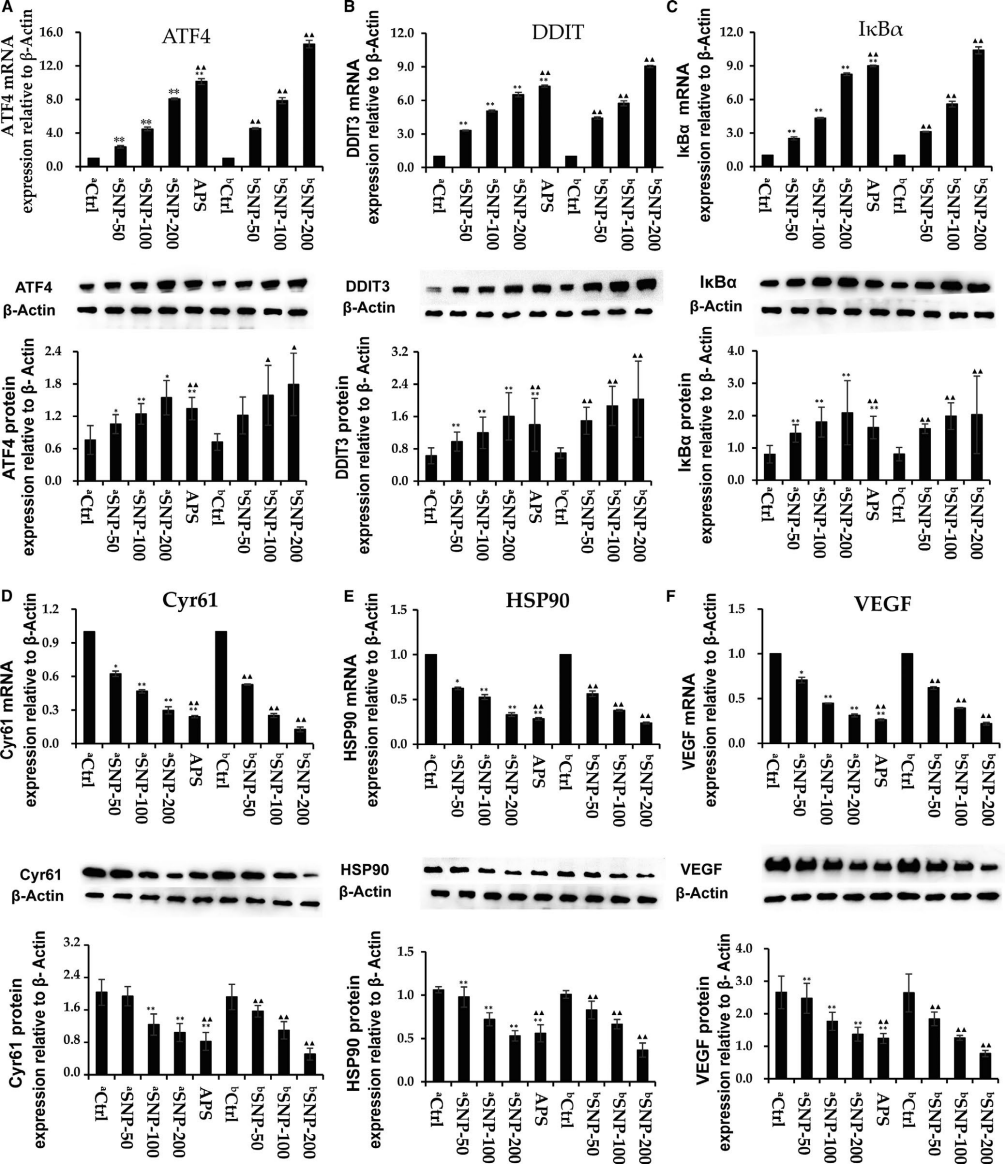
Effect of SNP on the mRNA and protein expression levels of activating transcription factor 4 (ATF4), DNA‐damage inducible transcript 3 (DDIT3), inhibitor of NF‐κB (IκBα), cysteine‐rich angiogenic inducer 61 (CYR61), HSP90, and vascular endothelial growth factor (VEGF) in HepG2 tumor‐bearing mice. Reverse‐transcription quantitative PCR and Western blotting were used to determine the expression levels of (A) ATF4, (B) DDIT3, (C) IκBα, (D) Cyr61, (E) heat shock protein 90 (HSP90), and (F) VEGF. a, therapeutic experiment; b, preventive experiment. Data are presented as the mean ± SD (*n* = 6). *p < 0.05, **p < 0.01 vs. aCtrl group; ^▲▲^ *p* < 0.05, ^▲▲^ *p* < 0.01 vs. bCtrl group. Ctrl, control; SNP, Sipunculus nudus polysaccharide; ATF4, activating transcription factor 4; DDIT3, DNA damage inducible transcript 3; CYR61, cellular communication network factor 1; HSP90, heat shock protein 90

The expression levels of several potential signalling proteins were measured in tumour samples to examine the antitumour mechanisms. According to previous studies, TNF‐α can regulate the protein kinase r‐like endoplasmic reticulum kinase (PERK)/eukaryotic initiation factor 2α (elF2α)/activating transcription factor 4 (ATF4)/the transcription factor C/EBP homologous protein (CHOP) axis,^18^ and ATF4 subsequently activates DNA damage–inducible transcript 3 (DDIT3).^19^ TNF‐α can influence inhibitor of NF‐κB (IκBα)*–*mediated signalling in inflammation.^20^ Here, we examined the gene and protein expression levels of ATF4, DDIT3 and IκBα (Figure 7A‐C). Compared with the ^a^Ctrl and ^b^Ctrl groups, the mRNA and protein expression levels of ATF4, DDIT3 and IκBα in tumour were significantly increased in all SNP‐related groups (*p* < 0.05), except for ATF4 protein expression in the ^b^SNP‐50 group (*p* > 0.05). We found that the mRNA and protein expression levels of ATF4, DDIT3 and IκBα in the ^b^SNP‐200 group were significantly higher compared with those in the APS‐positive group. However, the mRNA expression levels in the ^a^SNP‐200 group did not reach those of the APS‐positive group, but the same levels of protein expression were achieved. These results suggested that the mechanism of SNP’s immuno‐enhancement function may occur in part via the TNF‐α/ATF4/DDIT3 and TNF‐α/IκBα immunomodulatory signalling pathways, but the strength of the effect was different in therapeutic and preventive experiments.

Using a high‐throughput sequencing assay, we identified that the gene expression levels of Cyr61, Hsp90 and vascular endothelial growth factor (VEGF) were decreased in all SNP‐related groups (data not shown). Th mRNA and protein expression levels of cysteine‐rich angiogenic inducer 61 (Cyr61), heat‐shock protein 90 (Hsp90) and VEGF were detected (Figure 7D–F) and found to be downregulated in all SNP‐related groups in a dose‐dependent manner. The results suggested that the ^a^SNP‐50 and ^b^SNP‐100 groups were most similar to the APS‐positive group. According to these results, we concluded that the mechanism of SNP antitumour function may be exerted, at least partially, by blocking the actions of CYR61, HSP90 and VEGF.

### 3.9 SNP regulates the expression levels of apoptosis‐related proteins (caspase‐3, caspase‐8, caspase‐9, Bax, Bcl‐2 and VEGF)

**FIGURE 8:**
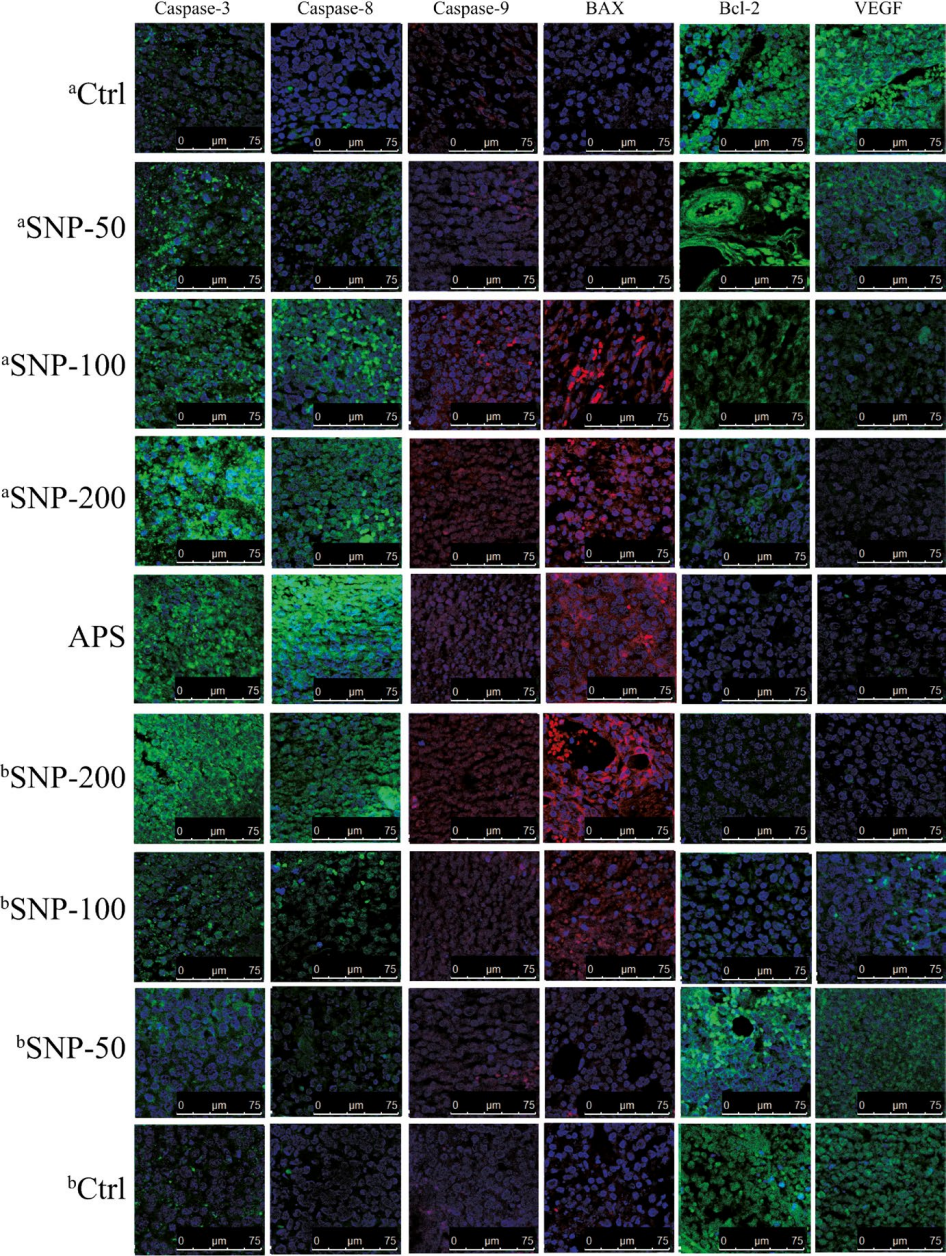
Effect of the SNP on the expression levels of caspase‐3, caspase‐8, caspase‐9, BCL2‐associated X (Bax), B‐cell lymphoma‐2 (Bcl‐2) and VEGF in HepG2 tumour‐bearing mice. An immunofluorescence staining assay was conducted to measure the expression levels of these factors. Blue staining represents the location of the nucleus. The intensity of green fluorescence or red fluorescence represents the expression level of different apoptotic proteins in tumour tissue. aCtrl, vehicle control group in the therapeutic experiment. aSNP‐50, aSNP‐100, and aSNP‐200 groups represent SNP treatment at concentrations of 50, 100 and 200 mg/kg, respectively, in the therapeutic experiment. bCtrl, vehicle control group in the preventive experiment. bSNP‐50, bSNP‐100, and bSNP‐200 groups represent SNP preconditioning at concentrations of 50, 100 and 200 mg/kg, respectively, in the preventive experiment. APS, the positive control group. Ctrl, control; SNP, Sipunculus nudus polysaccharide; the scale is 0–75 μM

It was reported that low VEGF levels downregulated the antiapoptotic factor B‐cell lymphoma‐2 (Bcl‐2),^21^ but upregulated the pro‐apoptotic factor BCL2‐associated X (Bax).^22^ The expression levels of apoptosis‐related proteins (caspase‐3, caspase‐8, caspase‐9, Bax, Bcl‐2 and VEGF) were detected via immunofluorescence analysis to investigate the antitumour mechanism of SNP. The relative expression levels of these proteins were analysed by comparing the fluorescence intensities in the tumour tissues of different groups (Figure 8). Blue staining represents the location of the nucleus. The intensity of green fluorescence or red fluorescence represents the expression level of different apoptotic proteins in tumour tissue. Compared with the ^a^Ctrl and ^b^Ctrl groups, the expression levels of caspase‐3, caspase‐8, caspase‐9 and Bax were notably increased in all SNP‐related groups and were enhanced to the same extent as in the APS‐positive group. However, the expression levels of Bcl‐2 and VEGF were markedly suppressed. These results suggested that SNP induced apoptosis in tumour cells.

## 4 DISCUSSION

A novel polysaccharide was extracted from *Sipunculus nudus* (SNP). The molecular weight (MW) of SNP was 9223 D according to HPGPC analyses, and the structure of SNP repeat units was composed of →3,4)‐β‐D‐GlcpNAC (1→ and →4) ‐α‐D‐Glcp (1→ in the ratio of 15:1; →2) ‐α ‐D‐Galp ‐ (1→ as the side chain; and β‐D‐Galp‐(1→ and α‐ D‐Glcp ‐ (1→ as the end group as determined by GC‐MS and NMR assays.

In our previous study, the flow cytometry assay was used to validate the pro‐apoptosis activity of SNP on HepG2.2.15 cells.^8^ Another study also reported the different potential immunologic effects of SNP.^6^ We identified several possible mechanisms to explain the antitumour activity of SNP on hepatoma HepG2‐bearing mice, which involves immunologic function.

The transepithelial electric resistance (TEER) of Caco‐2 cells shows that polysaccharide could be absorbed by membrane transport.^23^ Polysaccharide extracted from *Astragalus membranaceus* (APS) was found to exert a marked inhibitory effect on human solid tumours by potentiating the immune system, inducing apoptosis and inhibiting cancer cell growth.^24^ APS exerted a therapeutic effect on hepatocellular carcinoma H22‐bearing mice.^25^ APS could upregulate the level of nitric oxide (NO) and tumour necrosis factor‐α (TNF‐α), which acted as inducers of tumour cell apoptosis.^26^ So, APS was selected as the positive control in the present study. The tumour inhibition rates of SNP on hepatoma HepG2‐bearing mice were similar to those of the APS group. The thymus and spleen indices are considered to reflect the immunological function of the organism.^27^ In the present study, the thymus and spleen indices were significantly increased, and the morphological changes were evident in the tumour tissue after treatment with SNP, thus suggesting that SNP may inhibit HepG2 tumours via an enhanced immunological effect.

Cytokines and molecular pathways play pivotal roles in the immunomodulatory activity of organisms and in tumour development, and cytokines are the essential immunological response components that implement the antitumour function.^28^, ^29^ IL‐2 is an essential immunomodulatory cytokine that reflects cellular immunity; thus, an increase in IL‐2 level suggests the enhancement of cellular immunity in patients with cancer.^27^, ^29^ In the present study, IL‐2 levels were significantly increased in all SNP‐related groups, which indicated that SNP enhanced the immune function. IFN‐γ is a critical cytokine having both negative and positive immunomodulatory effects under different circumstances.^30^ A role of IFN‐γ for tumour rejection has been demonstrated by its cytotoxic activity on some tumour cells, upregulating MHC expression and thereby increasing tumour cell recognition and elimination, inducing expression of angiogenesis inhibitors, like IP‐10, by tumour cells.^31^ TNF‐α is another pleiotropic cytokine that was initially described as antitumorigenic.^32^ TNF‐α could naturally be bound to TNF receptor on cell surface, thereafter activate TRADD (the dead region protein), phosphorylate to JNK protein, increase the expression of p53 transcription factor and thus promote the transcription of BAX gene.^33^, ^34^ *Ganoderma lucidum* polysaccharides (GL‐PS) increased the concentration of serum IL‐2, TNF‐α and IFN‐γ and enhanced the cytotoxic activity of natural killer cells and T cells, inhibited glioma growth and prolonged the survival of rats.^35^ In the present study, the IFN‐γ and TNF‐α levels were significantly upregulated after SNP treatment in mice (especially at the concentrations of 100 and 200 mg/kg), indicating that SNP could strengthen the immune response by promoting the expression levels of IFN‐γ and TNF‐α.

In the endoplasmic reticulum (ER) stress response, TNF‐α exerts its role via the PERK–elF2α–ATF4–CHOP axis.^36^ ATF4 can activate DDIT3 (CHOP) activity and inhibit Bcl‐2/Bcl‐xl activity, ultimately promoting cell apoptosis.^37^ In inflammation and cancer, TNF‐α inhibits the activity of PPARγ via IκBα‐mediated signalling.^38^ IκBα usually exists with nuclear factor kappa‐B (NF‐κB) as heterodimers in the cytoplasm. When IκBα is separated from the heterodimer, NF‐κB is activated and then enters the nucleus to bind to DNA, which promotes the transcription of cytokines.^39^ In the present study, both the mRNA and protein expression levels of ATF4, DDIT3 and IκBα were markedly increased after SNP treatment in mice, suggesting that the SNP may be involved in two immunomodulatory signalling pathways to inhibit HepG2 tumour progression: the TNF‐α/ATF4/DDIT3 and TNF‐α/IκBα/PPARγ signalling pathways.

The present findings demonstrated that the gene expression levels of Cyr61, Hsp90 and VEGF decreased after SNP treatment, as detected using high‐throughput sequencing assay (data not shown). Cyr61 is associated with cell survival, proliferation and differentiation and is usually upregulated in human tumours.^40^ The overexpression of Cyr61 can activate mitogen‐activated protein kinase (MAPK) signalling and inhibit p53 activity,^41^ as well as promote vascular proliferation by upregulating VEGF.^42^ Usually, growth factor receptors, such as EGFR, or signal transduction proteins, including PI3K and AKT, are upregulated in tumour cells,^43^, ^44^ which are stabilized by HSP90.^45^ HSP90 can induce the expression of VEGF.^46^ It was reported that Hsp90^47^ and VEGF^48^ are important targets for anticancer therapeutics administered over a long time period. In the present study, the protein expression levels of CYR61, HSP90 and VEGF decreased after SNP treatment in mice (except at an SNP concentration of 50 mg/kg). Therefore, we concluded that SNP suppressed tumour growth in HepG2 tumour‐bearing mice by inhibiting the expression levels of CYR61, HSP90 and VEGF.

Along with the aforementioned signal molecules, ATF4 inhibits Bcl‐2/Bcl‐xl activity, thus promoting cell apoptosis.^37^ HSP90 can also modulate tumour cell apoptosis via AKT, TNFR and NF‐κB functions.^49^, ^50^ VEGF at a low level can reduce Bcl‐2, increase the Bax/Bcl‐2 ratio and enhance the expression of the Bax oligomer.^51^ Once the Bax oligomer binds to the mitochondrial membrane, the permeability of the mitochondrial membrane can be enhanced, which causes the translocation of Cyt‐C from the mitochondria to the cytoplasm.^52^ In the cytoplasm, the apoptotic body, formed from Apaf‐1, Cyt‐C and pro‐caspase‐9, induces the hydrolytic activation of caspase‐9, which can cause a cascade reaction and cleavage of cell DNA, ultimately leading to cell apoptosis.^53^ Pollen polysaccharides from Chinese wolfberry (WPPs) decreased the levels of AKT, p‐AKT and Bcl‐2 proteins and increased expression of Bax, caspase‐3 and caspase‐9 in DU145 cells, which eventually promoted apoptosis.^4^ In the current study, SNP significantly promoted the protein expression levels caspase‐3, caspase‐8, caspase‐9 and Bax in a dose‐dependent manner in tumour tissues of mice. Contrary to the pro‐apoptotic factors, Bcl‐2 and VEGF protein expression levels were inhibited. These results demonstrated that SNP induced apoptosis via the mitochondrial apoptosis pathway in tumour cells.

## 5 CONCLUSIONS

In conclusion, a novel polysaccharide was extracted from *Sipunculus nudus* (SNP). The molecular weight (MW) of SNP was 9223 Da according to HPGPC analysis, and the structure of SNP repeat units was determined by GC‐MS and NMR assays. The findings suggested that SNP exerts its antitumour activity by influencing immunoregulation and inducing the mitochondrial apoptosis of tumour cells. Thus, SNP may be investigated in the future as a promising candidate as an antitumour drug.

## 6 INSTITUTIONAL REVIEW BOARD STATEMENT

The experiments were performed in accordance with the guidelines of the Animal Care and Use Committee and Ethics Committee of Xiamen University. This study was approved by the Animal Ethics Committee of Xiamen University (Approval date: 13 March 2017). Human HepG2 cells were obtained from Shanghai MeiXuan Biological Technology Co., Ltd. The STR profile analysis indicated that the HepG2 cells were hepatoblastoma cells.

## CONFLICT OF INTEREST

The authors have declared that they have no competing interests.

## AUTHOR CONTRIBUTIONS

**jie su:** Conceptualization (equal); Data curation (equal); Formal analysis (equal); Funding acquisition (equal); Investigation (equal); Writing‐review & editing (equal). **Dengyuan Liao:** Formal analysis (equal). **Yongchang Su:** Methodology (equal). **Shuji Liu:** Methodology (equal). **Linlin Jiang:** Methodology (equal). **Jingna Wu:** Methodology (equal). **Zhiyu Liu:** Resources (equal). **Yuping Wu:** Investigation (equal); Supervision (equal).

## Supporting information

Supplementary Material

## Data Availability

Data are available in a publicly accessible repository.
