# Supplementary material for: Novel polysaccharide extracted from Sipunculus nudus inhibits HepG2 tumour growth in vivo by enhancing immune function and inducing tumour cell apoptosis
Source: J Cell Mol Med. 2021 Jul 24;25(17):8338–51. doi: 10.1111/jcmm.16793 (PMC8419178; doi:10.1111/jcmm.16793)
Supplement: Supplementary file 1 — Supplementary Material [file JCMM-25-8338-s001.docx]

Table S1. Sequences of primers used in real-time PCR analysis.

| Gene | Primer sequences |
| --- | --- |
| ATF4 F | TCCGAATGGCTGGCTGTGG |
| ATF4 R | AGTGTAGTCTGGCTTCCTATCTCC |
| DDIT3 F | AGCAGAGGTCACAAGCACCT |
| DDIT3 R | CTGGGGAATGACCACTCTGT |
| CYR61 F | ATTGGATCCATGAGCTCCCGCATCGCC |
| CYR61 R | CGGGAATTC TTAGTCCCTAAATTTGTG |
| IκBα F | GACGAGGATTACGAGCAGAT |
| IκBα R | CCTGGTAGGTTACTCTGTTG |
| VEGF F | CTACCTCCACCATGCCAAGT |
| VEGF R | AGCTGCGCTGATAGACATCC |
| β-Actin F | ctgggacgacatggagaaaa |
| β-Actin R | aaggaaggctggaagagtgc |

Table S2. Results of SNP methylation hydrolysis by GC-MS

| Sample | Connection | Derivative | MW | Relative molar ratio (%) |
| --- | --- | --- | --- | --- |
| SNP | t-Fuc(p) | 1,5-di-*O*-acetyl-6-deoxy-2,3,4-tri-*O*-methyl fucitol | 293 | 0.428 |
|  | t-Xyl(p) | 1,5-di-*O*-acetyl-2,3,4-tri-*O*-methyl xylitol | 279 | 2.977 |
|  | t-Man(p) | 1,5-di-*O*-acetyl-2,3,4,6-tetra-*O*-methyl mannitol | 323 | 6.549 |
|  | t-Glc(p) | 1,5-di-*O*-acetyl-2,3,4,6-tetra-*O*-methyl glucitol | 323 | 15.534 |
|  | t-Gal(p) | 1,5-di-*O*-acetyl-2,3,4,6-tetra-*O*-methyl galactitol | 323 | 38.592 |
|  | 1,4-Fuc(p) | 1,4,5-tri-*O*-acetyl-6-deoxy-2,3-di-*O*-methyl fucitol | 321 | 0.567 |
|  | 1,2-Xyl(p) | 1,2,5-tri-*O*-acetyl-3,4-di-*O*-methyl xylitol | 307 | 2.700 |
|  | 1,2-Man(p) | 1,2,5-tri-*O*-acetyl-3,4,6-tri-*O*-methyl mannitol | 351 | 1.482 |
|  | 2,3-Fuc(p) | 1,2,3,5-tetra-*O*-acetyl-6-deoxy-4-*O*-methyl fucitol | 349 | 3.747 |
|  | 1,2-Gal(p) | 1,2,5-tri-*O*-acetyl-3,4,6-tri-*O*-methyl galactitol | 351 | 20.307 |
|  | 1,6-Glc(p) | 1,5,6-tri-*O*-acetyl-2,3,4-tri-*O*-methyl glucitol | 351 | 1.336 |
|  | 1,4-Glc(p) | 1,4,5-tri-*O*-acetyl-2,3,6-tri-*O*-methyl glucitol | 351 | 3.008 |
|  | 1,6-Gal(p) | 1,5,6-tri-*O*-acetyl-2,3,4-tri-*O*-methyl galactitol | 351 | 0.750 |
|  | 2,4-Gal(p) | 1,2,4,5-tetra-*O*-acetyl-3,6-di-*O*-methyl galactitol | 379 | 0.383 |
|  | 3,6-Man(p) | 1,3,5,6-tetra-*O*-acetyl-2,4-di-*O*-methyl glucitol | 379 | 0.940 |
|  | 2,6-Gal(p) | 1,2,5,6-tetra-*O*-acetyl-3,4-di-*O*-methyl galactitol | 379 |  |

Table S3 Assignment of ^1^H and ^13^C chemical shifts of sugar residues in SNP

| Sugar residues | | chemical shift（ppm） | | | | | | | | | |
| --- | --- | --- | --- | --- | --- | --- | --- | --- | --- | --- | --- |
|  |  |  | 1 | 2 | 3 | 4 | 5 | 6a/6b | CH3 of NAC | CO fo NAC |  |
| A | β-D-Galp-(1→ | H | 4.34 | 3.56 | 3.68 | 3.96 | 3.66 | 3.77/-- |  |  |  |
|  |  | C | 105.86 | 75.40 | 75.50 | 72.19 | 74.24 | 63.43 |  |  |  |
| B | →2)-α-D-Gal*p*-(1→ | H | 5.23 | 3.91 | 4.09 | 3.93 | 3.51 | 3.68 |  |  |  |
|  |  | C | 98.81 | 75.88 | 71.11 | 71.89 | 71.60 | 63.40 |  |  |  |
| C | α-D-Glcp-(1→ | H | 4.87 | 3.47 | 3.66 | 3.32 | 3.88 | 3.75 |  |  |  |
|  |  | C | 100.95 | 71.60 | 72.19 | 72.27 | 69.17 | 63.30 |  |  |  |
| D | →4)-α-D-Glcp-(1→ | H | 5.33 | 3.44 | 3.97 | 3.65 | 3.34 | 3.69 |  |  |  |
|  |  | C | 100.33 | 69.55 | 71.60 | 76.11 | 71.81 | 63.45 |  |  |  |
| E | →3,4)-β-D-GlcpNAc(1→ | H | 4.55 | 3.74 | 3.55 | 3.44 | 3.42 | 3.99 | 2.02 |  |  |
|  |  | C | 104.18 | 57.40 | 80.31 | 83.33 | 73.36 | 63.42 | 25.14 | 175.4 |  |


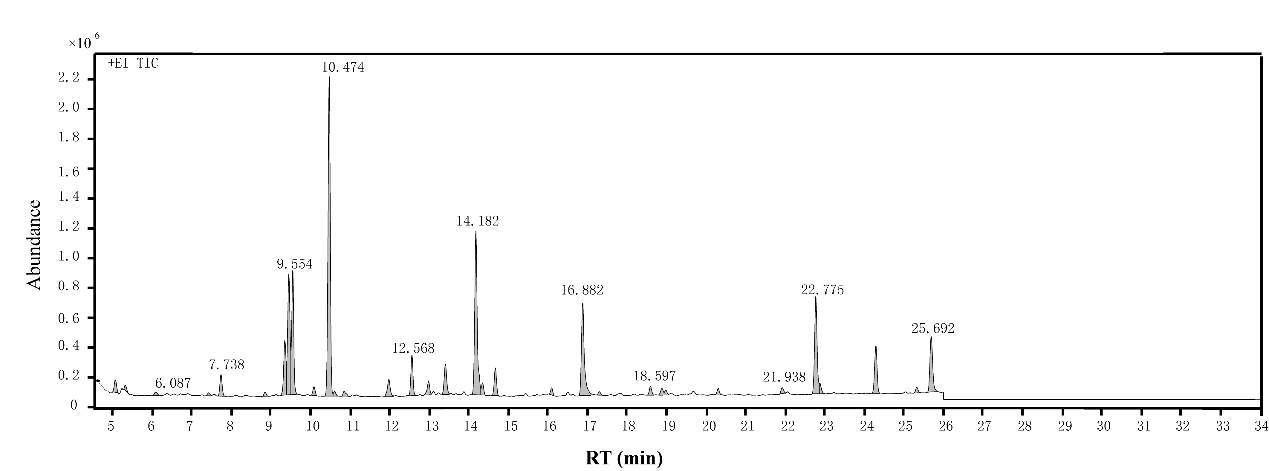


Figure S1. Total ion flow diagram of SNP


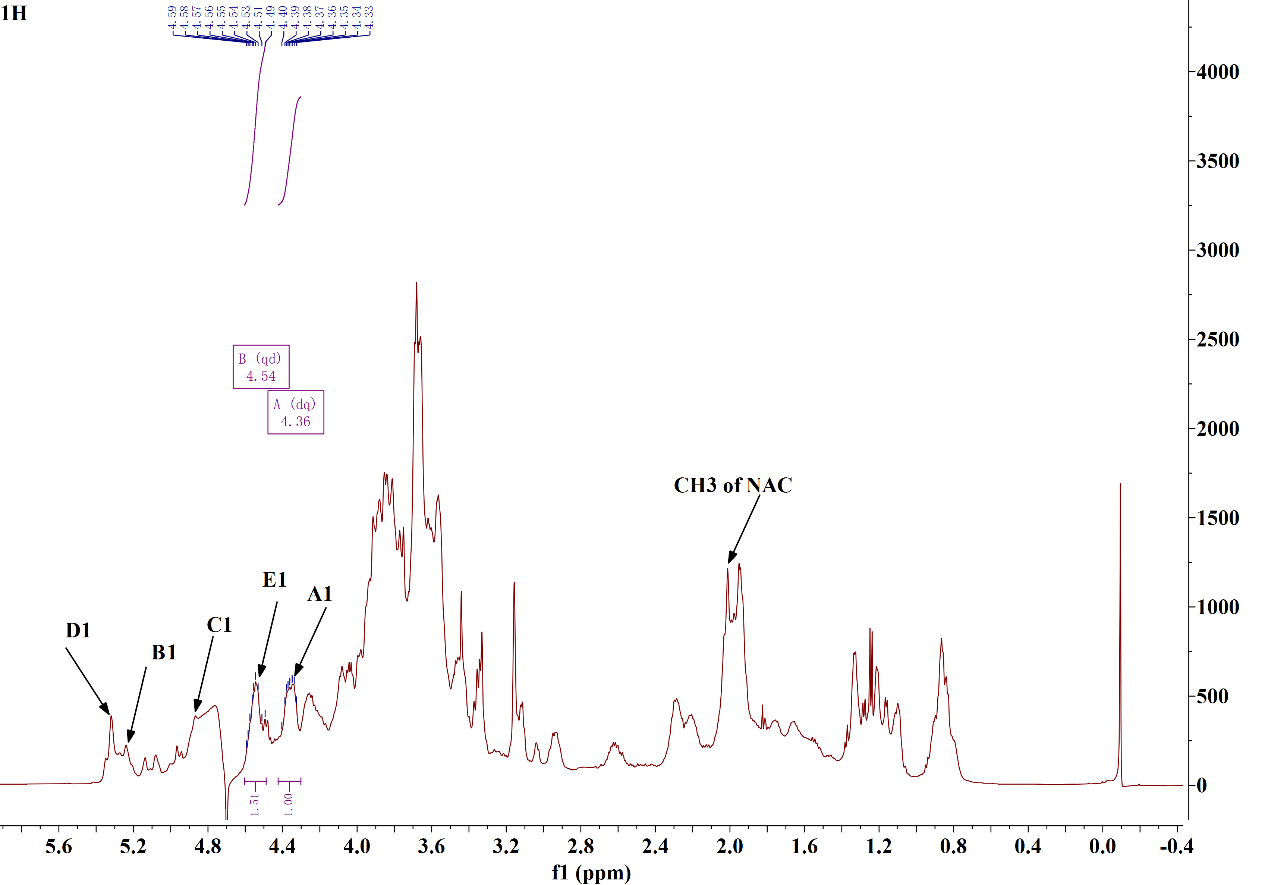


Figure S2. H-spectrum of SNP


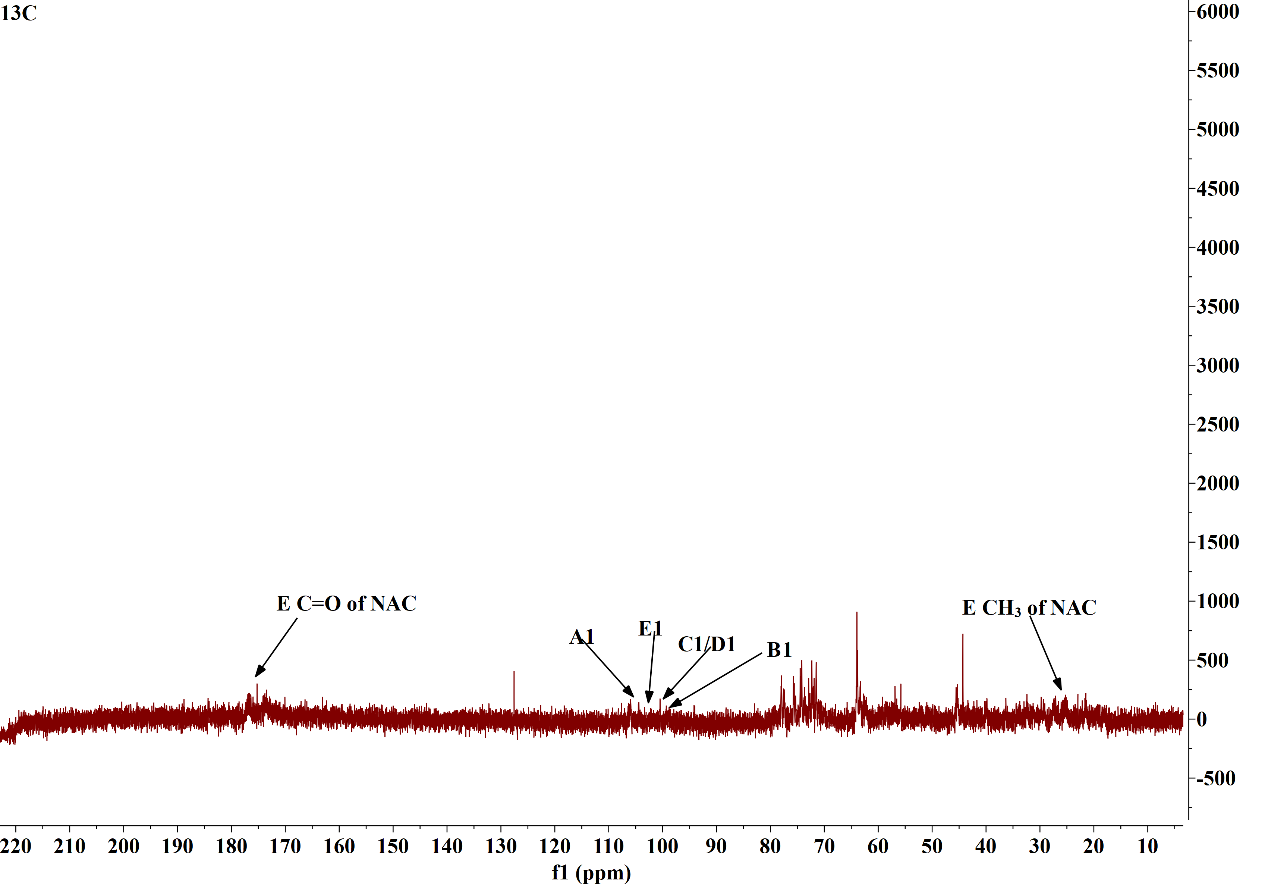


Figure S3. C-spectrum of SNP


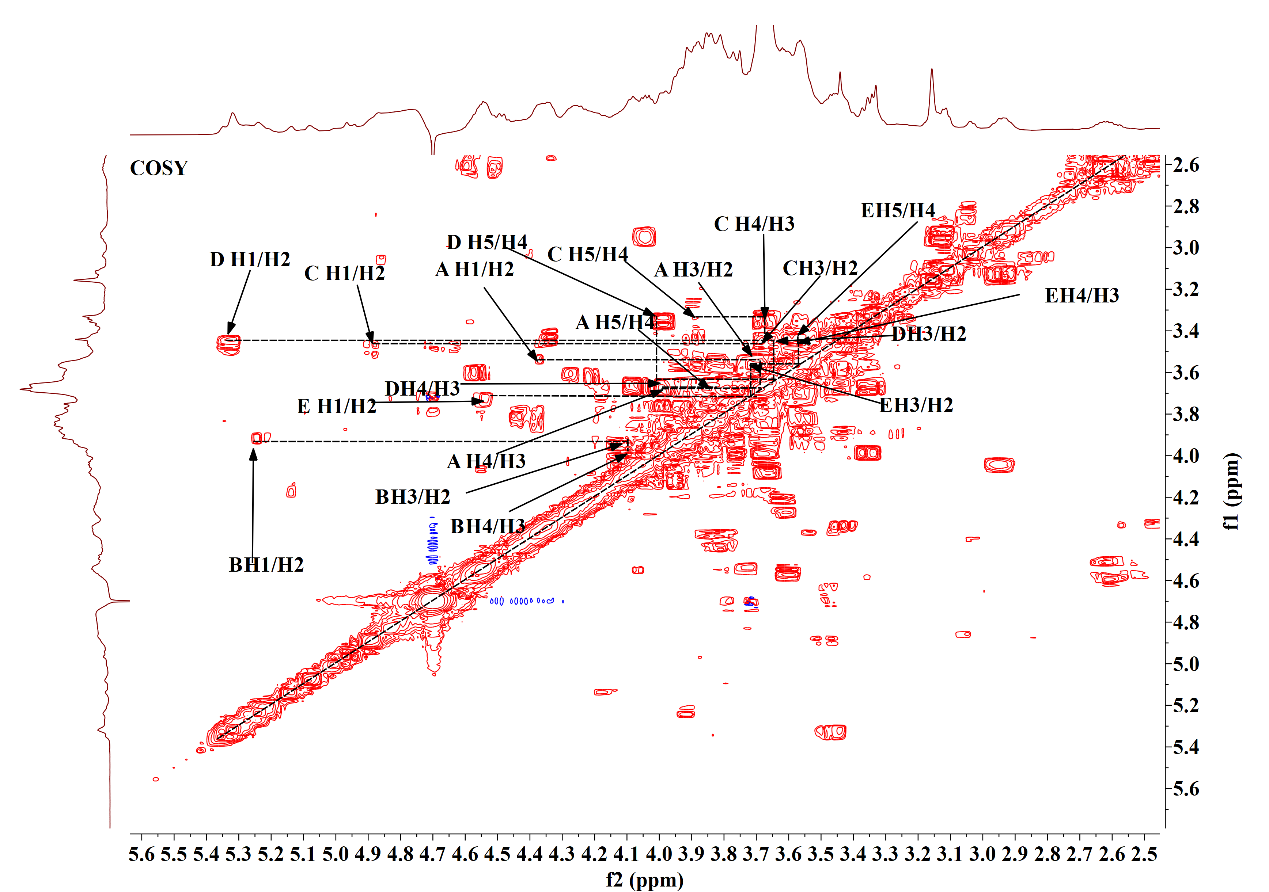


Figure S4. COSY spectrum of SNP


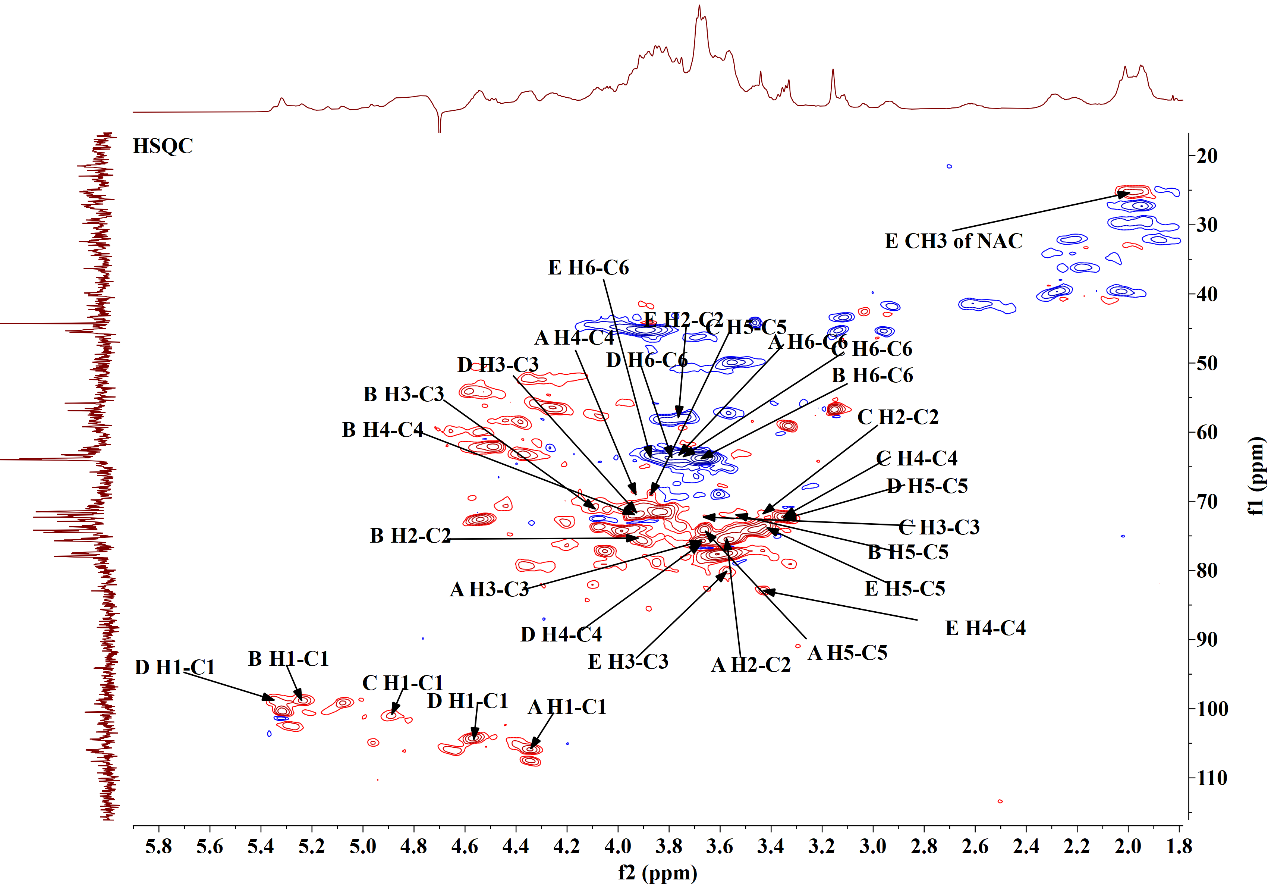


Figure S5 HSQC spectrum of SNP


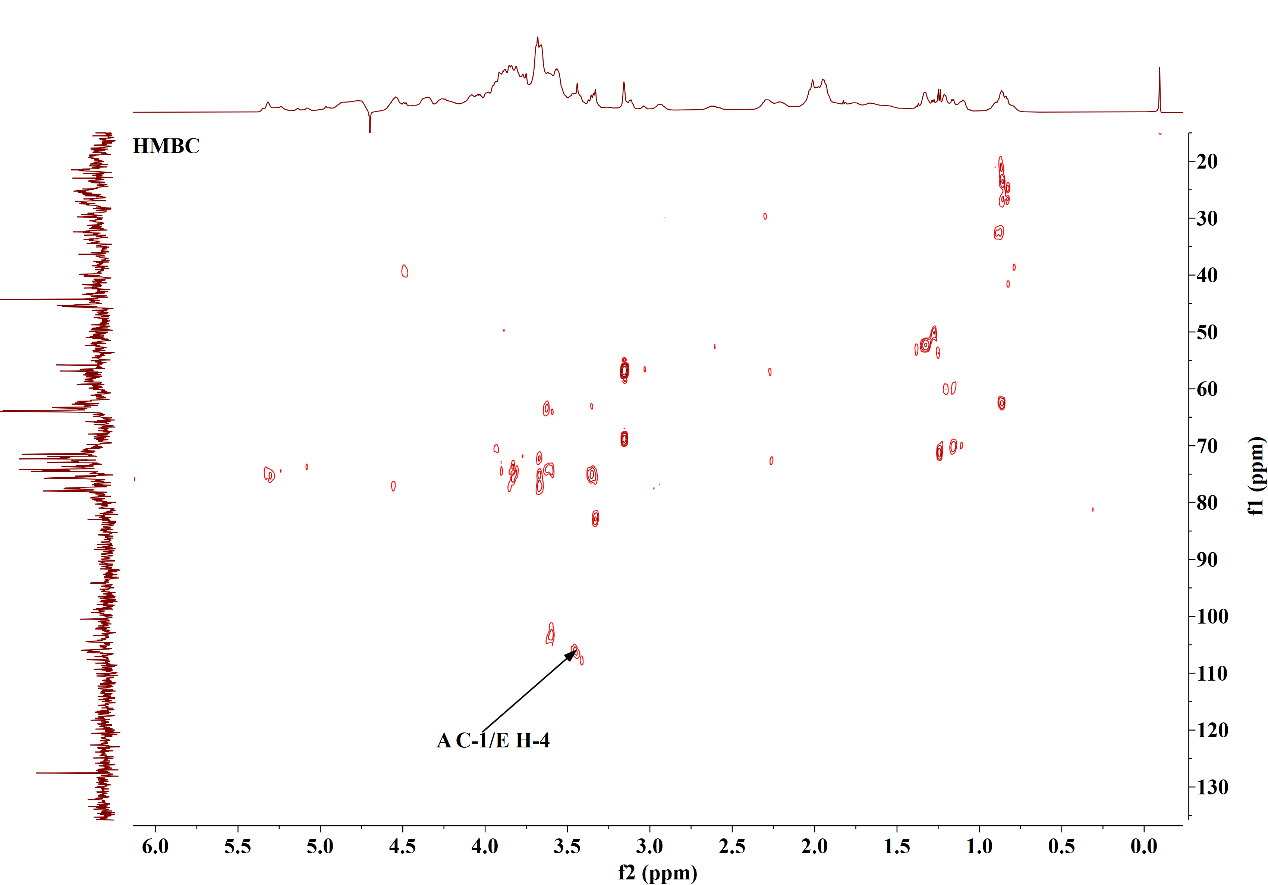


Figure S6. HMBC spectrum of SNP


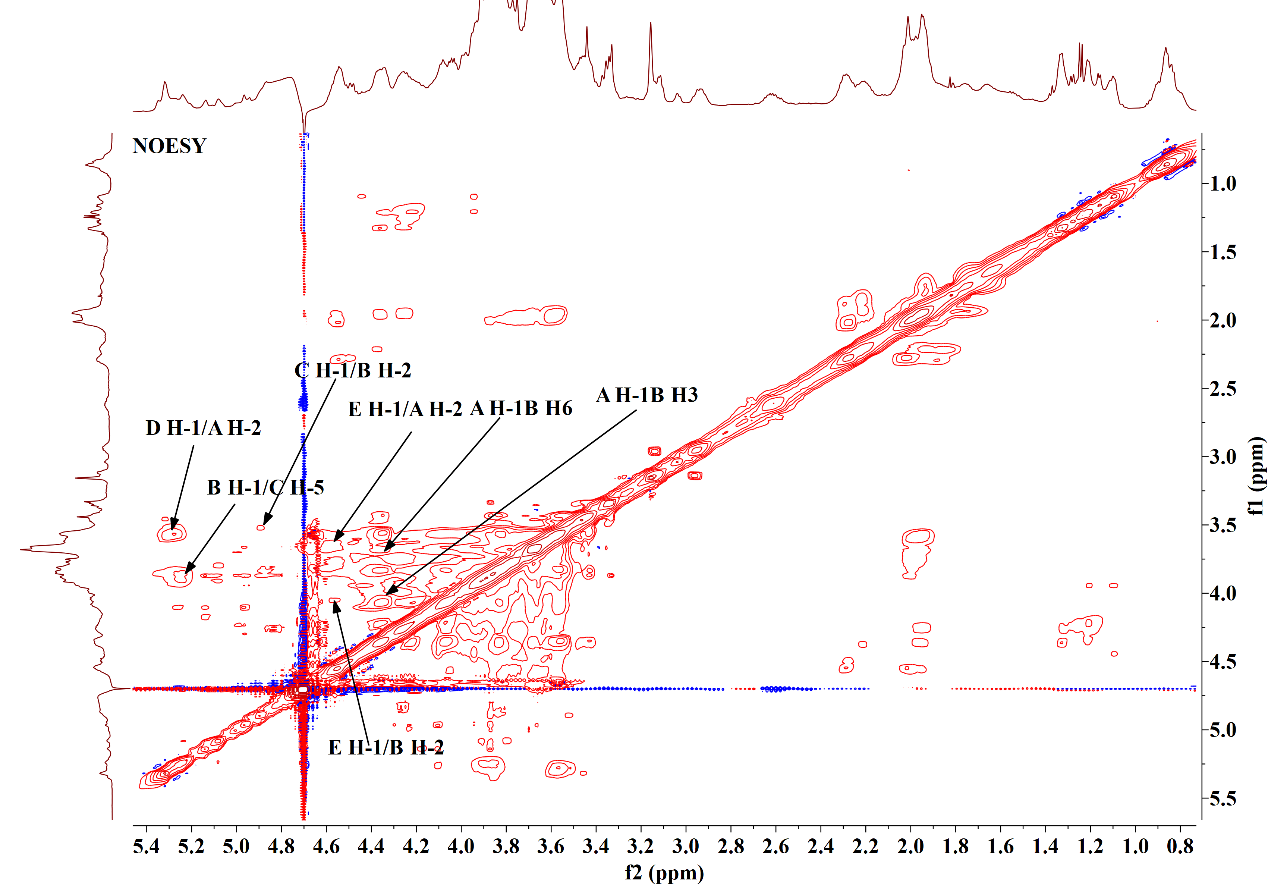


Figure S7. NOESY spectrum of SNP
